# Improving the Conductivity of the PEDOT:PSS Layers in Photovoltaic Cells Based on Organometallic Halide Perovskites

**DOI:** 10.3390/ma15030990

**Published:** 2022-01-27

**Authors:** Yuliya Spivak, Ekaterina Muratova, Vyacheslav Moshnikov, Alexander Tuchkovsky, Igor Vrublevsky, Nikita Lushpa

**Affiliations:** 1 Saint Petersburg Electrotechnical University “LETI”, 197376 Saint Petersburg, Russia; ymkanageeva@yandex.ru (Y.S.); vamoshnikov@mail.ru (V.M.); 2 R&D Laboratory of Materials and Components of Electronics and Superconducting Equipment, Belarusian State University of Informatics and Radioelectronics, 220013 Minsk, Belarus; tak53@mail.ru (A.T.); vrublevsky@bsuir.edu.by (I.V.); lushpa@bsuir.by (N.L.)

**Keywords:** organometallic perovskites, photovoltaic applications, grain boundaries, amines, p-type doping, carbon nanotubes, electrical conductivity

## Abstract

Among conductive polymers, PEDOT films find the widest application in electronics. For photovoltaic applications, studies of their optical properties, stability, and electrical conductivity are of greatest interest. However, the PEDOT:PSS transport layers, when used in photovoltaic cells, have a high electrical resistance, which prevents solar cells from increasing their efficiency. One of the promising ways to improve their electrical properties is the use of composite materials based on them, in which the conductivity can be increased by introducing various additives. In this work, conductive polymer films PEDOT:PSS (poly (3,4-ethylenedioxythiophene):polystyrene sulfonate acid) doped with a number of amines (Pentylamine, Octylamine, Diethylamine, Aniline with carbon nanotubes) were obtained and studied. It is shown that, depending on the concentration of dopants, the electrical conductivity of PEDOT:PSS films can be significantly improved. In this case, the light transmission of the films practically does not change. The process of improving the conductivity by treating the surface of the finished film with amines, followed by heat treatment, was studied. It is assumed that the improvement in conductivity is the result of the self-assembly of monolayers of organic molecules on the surface of the PEDOT:PSS film leading to its *p*-doping due to intermolecular interaction.

## 1. Introduction

The development of environmentally friendly alternatives to existing traditional methods of generating electricity from coal and fossil fuels is essential to preserve the global environment and ensure sustainable economic growth. The widespread use of photovoltaic solar cells seems to be one of the most effective ways to overcome the energy crisis [1]. In 2019, photovoltaics provided almost 3% of the world’s electricity, while 15 years ago, it accounted for less than one-tenth of a percent; 2019 was the year of record global growth in photovoltaic capacity.

The sustainable development scenario for the period from 2019 to 2030 provides for their average annual growth of 15%. A relatively recent new but rapidly developing area is solar cells based on hybrid organic-inorganic semiconductor materials with a perovskite structure, such as (CH_3_NH_3_)PbX_3_ (X = Cl, Br, I) and their analogs [2,3,4]. This class of semiconductors has attracted a lot of attention due to its excellent photosorption characteristics. The combination of electrical properties and absorption characteristics of hybrid perovskites made it possible during 2012–2013 to increase the efficiency of energy conversion of solar cells based on them from 7.2% to 20%.

To date, it is recognized that polycrystalline films of organometallic halide perovskites with large crystallite sizes make it possible to achieve higher values of the conversion efficiency of light energy into electrical energy. The reason is a decrease in the area of intercrystalline boundaries, where traps of photogenerated charge carriers are mainly concentrated, and a long diffusion length of carriers, which makes it possible to use rather thick films in cells. In CH_3_NH_3_PbI_3_ single crystals grown from solution, the diffusion length can exceed 175 μm under 1 SUN illumination (100 mW/cm^2^) [5]. Internal quantum efficiency approaches 100% in monocrystalline perovskite solar cells. Such a long diffusion length is the result of higher carrier mobility, a longer lifetime, and a much lower density of traps in single crystals than in polycrystalline films [5]. The achieved efficiency of cells on a single crystal of 21.1% is also largely due to the very high value of the filling factor (94.3%) [6]. However, the formation of solar cells on grown single crystals for mass production is ineffective; therefore, it is natural for researchers to strive to obtain polycrystalline films with crystallites, the thickness of which is equal to the thickness of the polycrystalline film. In particular, a new growth method was proposed based on the asymmetric cavitation method of crystallization, which promotes heterogeneous nucleation, providing enough energy to overcome the crystallite nucleation barrier [7]. Various other methods were used to obtain films of perovskites with micron-sized crystallites, including those with highly oriented crystallites [8] since their disorientation in the film also impairs the transport properties of the film.

It should be noted that in the literature, there is not always unambiguous information about the influence of intercrystalline boundaries on various cell parameters [9]. Thus, there is no doubt that the grain size and intercrystalline boundaries, apparently, play an important role in the series resistance [10], the filling factor, and hysteresis [11], but their role is less in the recombination of carriers under typical operating conditions. It is believed that a small crystallite size in the range of less than 100 nm degrades the performance of devices. This is due to an increase in the path for the movement of ions and, therefore, a higher hysteresis. In any case, as confirmed by numerical simulations, the improved characteristics of solar cells are associated with a decrease in volume defects and improved mobility of charge carriers in coarse-crystalline films [12].

The grain boundaries in films act as solid walls that restrict the diffusion of carriers inside the film [13]. However, a limitation for the diffusion of carriers can be subcrystallite singular boundaries, which are not visible in an optical microscope but are manifested in the quenching of photoluminescence in the region of intercrystalline boundaries [14].

Thus, the main directions for improving the quality of perovskite films are increasing the continuity of the film surface coating on substrates, obtaining a large crystallite/grain size reducing the number of grain boundaries, etc. [12]. The large size of perovskite crystallites is an advantage for higher conversion efficiency of charge carriers due to fewer grain boundaries that increase electrical resistance, and where non-radiative losses and non-radiative recombination caused by traps are likely to occur [15].

Since the charge-transport layers of photovoltaic cells (PEDOT:PSS, transition metal oxides, Spiro-OMeTAD, etc.) do not differ in high electrical conductivity, it is necessary to find ways to increase the efficiency of the cells. One of these ways is the use of composite materials, in which the conductivity increases due to the inclusion of various additives in the material of the charge-transport layer.

Among conductive polymers, PEDOT (poly (3,4-ethylene dioxythiophene)) is most widely used in electronics, in particular in photovoltaics. PEDOT is unstable in its neutral state because it oxidizes rapidly in air. It is also insoluble in most solvents but can be dispersed in water using polystyrene sulfonated acid (PSS) as a counter ion. PSS also serves as an excellent oxidizing agent. However, in an aqueous dispersion of PEDOT:PSS, short PEDOT chains are surrounded by a thin PSS enriched with a surface layer, which is one of the main reasons for its low conductivity. In view of the importance of this material for electronics, a large number of scientific works and reviews are devoted to the study of its synthesis conditions, structure, and various properties (see, for example, the last review [16]). For photovoltaic applications, studies of its optical properties, stability, and ways of increasing electrical conductivity are of the greatest interest. It noticeably increases with increasing film thickness, but in solar cells, the thickness of the films is 30–60 nm.

Improving PEDOT:PSS conductivity reduces series resistance losses, which in turn leads to an increase in cell efficiency (PCE). This makes the material even more suitable as a transparent electrode material and thus results in better cell performance. PEDOT:PSS conductivity additives include high dielectric solvents such as dimethyl sulfoxide and N, N-dimethylformamide, or polar compounds such as glycerol, ethylene glycol, polyalcohols, and sorbitol [15,16,17,18,19,20]. The reason for the improvement in PCE and conductance is mainly due to morphological changes. In addition, with a conductivity exceeding 1000 S·cm^–1^, PEDOT:PSS approached the characteristics of ITO and is considered as an appropriate alternative for a convenient replacement for ITO in a number of applications, including as electrodes in solar cells with active organic materials [17,18,19]. For the same purpose, PEDOT:PSS is modified by treatment of various external exposure (ion irradiation, gamma-ray irradiation, UV irradiation) [21,22,23] or by additional processing using organic and inorganic acids, such as acetic acid, propionic acid, butyric acid, oxalic acid, sulfurous acid, and hydrochloric acid, etc. [24,25,26,27,28].

In this paper, the process of improving the conductivity by treating the surface of the PEDOT:PSS film with amines, followed by heat treatment, was studied. It is assumed that the improvement in conductivity is the result of the self-assembly of monolayers of organic molecules on the surface of the PEDOT:PSS film leading to its p-type doping due to intermolecular interaction.

## 2. Materials and Methods

In order to use PEDOT:PSS films as p-charge-transport layers, they must have good conductivity but a barrier for electrons. Conductivity in such polymer films passes through a system of conjugated double bonds. Pure PEDOT:PSS films have a very low conductivity, and the acidic nature of PEDOT:PSS degrades organic films (in particular perovskite) and causes chemical reactions at the layer boundaries, which leads to an increase in the density of traps and causes electrode corrosion. Therefore, its possible modification by a number of substances, primarily acids, is not acceptable for photovoltaic applications. In this regard, the idea of using amines to increase the conductivity of PEDOT:PSS seems to be attractive. Previously, they were used in solar energy for the synthesis of new organic p - conducting materials [29,30]. An aqueous solution of PEDOT:PSS (0.3 wt.% Sigma Aldrich, St. Louis, MO, USA) was used for the research.

The choice of a class of amines for the treatment of PEDOT:PSS films was based on the possibility of binding partially positively charged groups in amines with negatively charged sulfo groups of PSS. In this case, the amine ion attracts electrons to itself. This bonding leads to an improvement in the conjugation of double bonds in PEDOT. As a result, in such a system, p-type doping of PEDOT:PSS films occurs, leading to an improvement in conductivity. Moreover, it is assumed that the use of treated PEDOT:PSS films will lead to a decrease in their destructive effect on the properties of perovskites due to the neutralization of sulfo groups, which could affect the perovskite films.

**Figure 1:**
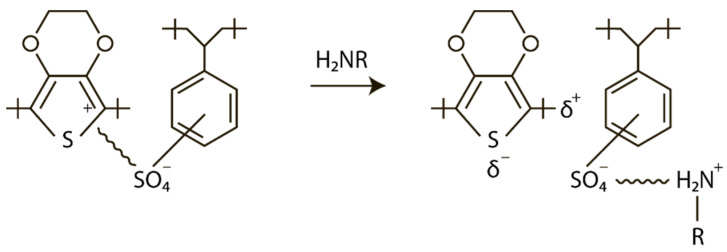
Scheme of the mechanism of intermolecular interaction of PEDOT:PSS with amines.

The following types of amines (Alfa Aesar, Haverhill, MA, USA) were used for investigation: Aniline, 99+% (C_6_H_7_N); Diethylamine, 99+% (C_4_H_11_N); Octylamine, 99 % (CH_3_(CH_2_)_7_NH_2_); 1-Pentylamine, 98% (CH_3_(CH_2_)_4_NH_2_); Methylamine, 40% aq. soln. (CH_3_NH_2_). The amine solution easily wets the surface of the PEDOT:PSS film, and then the amines selectively react with the PSS sulfo groups. The PEDOT material is not affected by this. Therefore, the PEDOT:PSS film remains homogeneous after treatment with amines. The mechanism of intermolecular interaction of PEDOT:PSS with amines upon their treatment with amine solutions is shown in Figure 1.

Two treatment options for PEDOT:PSS with amines were used. In the first case, a certain amount of amines was added to the aqueous solution of PEDOT:PSS. In the second case, the formed PEDOT:PSS film was treated with an amine solution of the appropriate concentration. 

**Figure 2:**
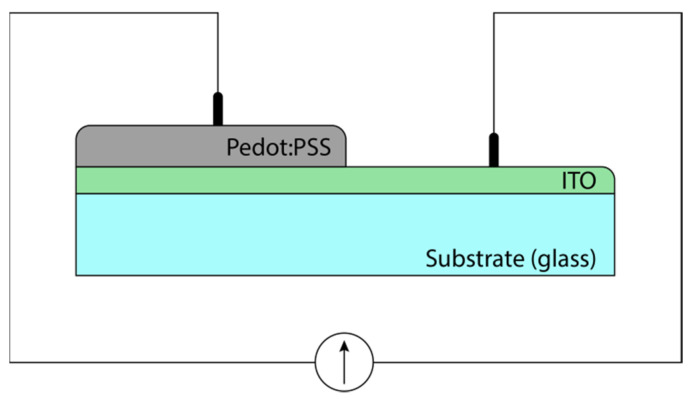
PEDOT:PSS film resistance measurement circuit.

The solution of PEDOT:PSS with amines was applied to glass at a temperature of 70–80 °C, followed by centrifugation. The films obtained had a thickness of about 100 nm. PEDOT:PSS film is a soft material, and the use of test probes for measuring electrical resistance resulted in scatter of data [31]. Therefore, for the samples, the electrical resistance was measured between plate electrodes located at a distance of 1 mm from each other (Figure 2).

In the second case, the surface of a freshly prepared PEDOT:PSS film was dried at a temperature of 105 °C to remove residual water. Then the film was treated with amine and re-dried to remove the solvent and enhance the chemical interaction. The film was then heat-treated.

A solution of multi-walled carbon nanotubes was prepared by adding an appropriate amount of them to water, followed by ultrasonic treatment for 30 min. The resulting stable homogeneous system of multi-walled carbon nanotubes was added to a PEDOT:PSS solution. The spectral transmission of composite films on glass substrates was measured in the range 400–800 nm (UV-3600 spectrophotometer, Shimadzu Corporation, Kyoto, Japan).

**Figure 3:**
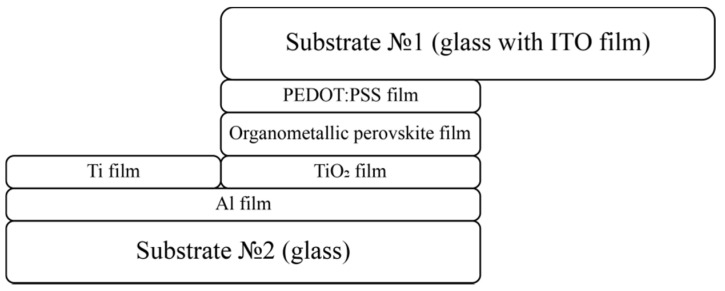
A scheme showing the perovskite-based solar cell structure.

In this work, a p-i-n structure was chosen for the manufacture of a photovoltaic cell based on perovskite. A schematic cross-sectional representation of such a structure with all layers is shown in Figure 3.

**Figure 4:**
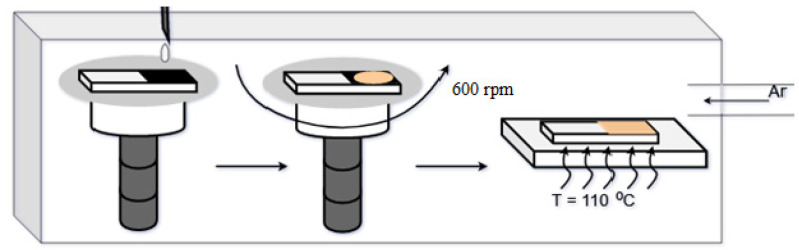
A scheme showing the process of applying N-methylpyrrolidone to a perovskite substrate.

Perovskite was prepared by mixing a solution of methylammonium iodide (CH_3_NH_3_I) and lead iodide (PbI_2_) in a polar organic solvent N-methylpyrrolidone (NMP). The resulting mixture was then stirred at 60 °C for 12 h. The perovskite solution obtained was applied by centrifugation onto a substrate heated to 110 °C (Figure 4).

The samples were then heat-treated at 110 °C for 30 min in an argon atmosphere.

## 3. Results and Discussion

**Figure 5:**
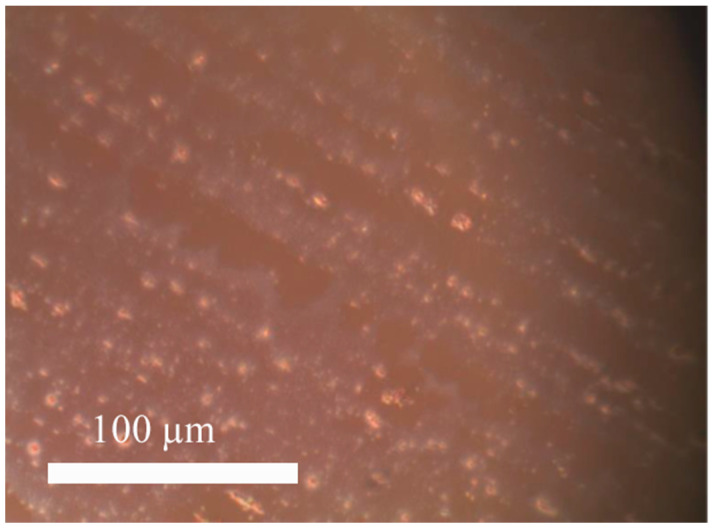
Photo of the surface of a glass substrate with a transparent electrode, a charge-transport layer (PEDOT:PSS), and perovskite microcrystallites after drying (optical microscope, magnification 500×).

As a result of the first stage of the formation of perovskites on the substrate after evaporation of the solvent, nanocrystallites with sizes of tens to hundreds of micrometers are formed (Figure 5). They are growth centers (seeds) at the second stage of film formation when a concentrated perovskite film precursor is applied to the substrate.

**Figure 6:**
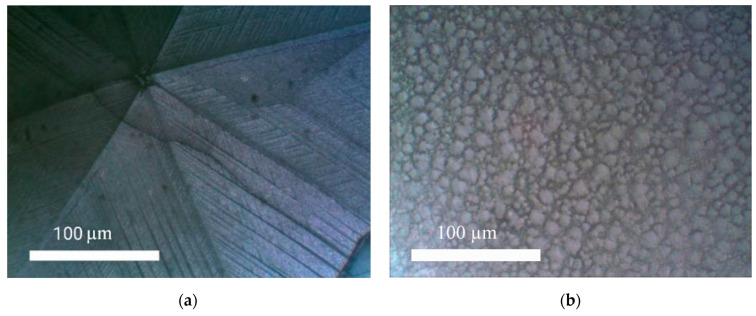
Photograph of the surface of films with large crystallites and small crystallites obtained by a two-stage method (optical microscope, magnification 500×: (**a**) the surface of films with large crystallites; (**b**) the surface of films with small crystallites.

As a result of the second stage, a perovskite film with large crystallite sizes was formed on the substrate. Photographs of the film surface are shown in Figure 6a. At lower substrate temperatures at the second stage (120 °C), many crystallites 1–20 µm in size formed on its surface (Figure 6b).

**Figure 7:**
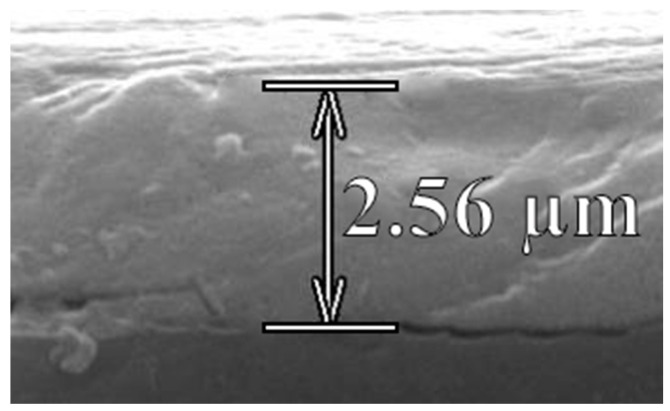
SEM image of Cross-section of a perovskite film obtained by a two-stage method.

In order to obtain images using a scanning electron microscope, the films were deposited on a glass substrate (Figure 7). Coarse-grained films have a fairly large thickness: in this case, the thickness values were in the range of 2.5–3.5 µm. This was to be expected since it is known that the growth of crystallites depends on the film thickness. The thicker the film, the larger the crystallites [32].

As SEM studies have shown, the films obtained by the two-stage method are distinguished by high homogeneity and the absence of punctures on the substrate area of ~1.0 cm^2^, compared to the usual several square millimeters. 

**Figure 8:**
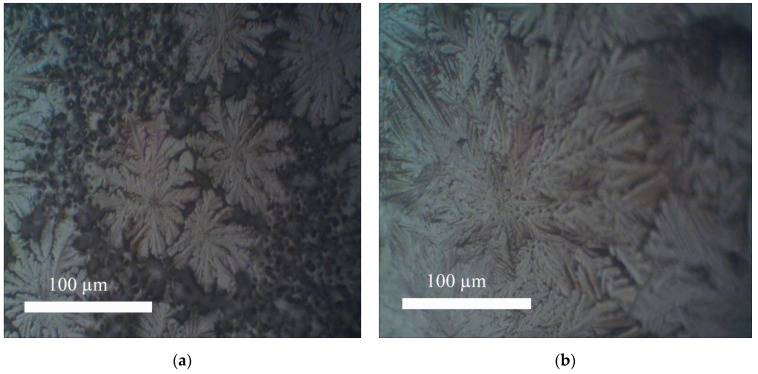
Dendrites on the surface of the CH_3_NH_3_PbI_3_perovskite film for different concentrations of the primary solution for the formation of crystallization centers (optical microscope, magnification 500×): (**a**) ~0.3%; (**b**) ~0.1%.

With a decrease in the concentration of the perovskite solution at the first stage of film preparation and a decrease in the substrate temperature, the film growth process radically changes. With the growth of the film at the second stage of the process, the formed crystallites deplete the concentration of the solution, and it falls below the minimum concentration of nucleation. At a higher substrate temperature and rapid evaporation of the solvent, the perovskite concentration would remain high, and the crystallite growth process would then continue [33]. Therefore, the growth of crystallites begins on the surface, where, due to the evaporation of the solvent, the perovskite concentration is higher. As a result, a dendritic structure forms on the surface [34,35]. The dendritic structure formed in our case is shown in Figure 8. 

**Figure 9:**
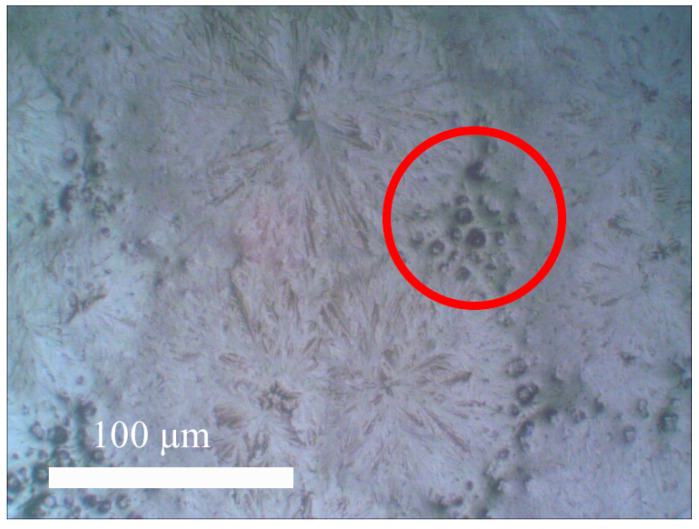
The surface of the CH_3_NH_3_PbI_3_ perovskite film with the addition of multi-walled CNT (optical microscope, magnification 500×).

It is interesting that the size of the dendrites depends on the concentration of the solution at the first stage of the film formation process. The smaller it is, the larger the size of the dendrites (Figure 8). At a higher concentration (> 1%), dendrites are not formed. The thickness of all films is 2 microns. Films of perovskite CH_3_NH_3_PbI_3_ with the addition of multi-walled CNTs were also formed. The surface of the films observed with an optical microscope is shown in Figure 9.

It can be seen from the Figures that the tubes are embedded in the space between the crystallites in the form of peculiar conglomerates. This should lead to a decrease in the loss of charge moving between the electrodes of the photovoltaic cell. Indeed, as the measurements showed, with an increase in the CNT concentration to 0.1 mass%, the resistance of the perovskite film nonlinearly decreased by more than 1.5 times. This indicates that the charge-transport and adhesive properties of PEDOT:PSS layers of photovoltaic cells can be improved using amines.

**Figure 10:**
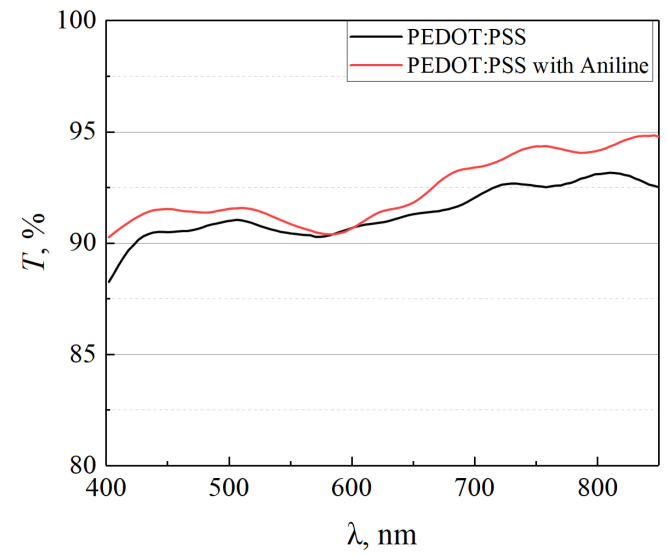
The transmission spectra of original PEDOT:PSS film and such film modified with aniline on glass substrates.

The spectral transmission of the original PEDOT:PSS film and such film modified with aniline on glass substrates are shown in Figure 10.

As it can be seen from Figure 10, the treatment of the PEDOT:PSS film with aniline leads to a slight increase in transparency. This may be related to a narrowing of the bandgap width due to the weakening of chemical bonds.

**Figure 11:**
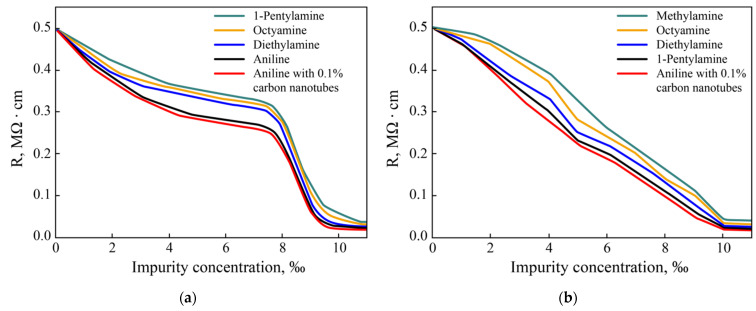
Change in the resistivity of the PEDOT:PSS films with (**a**) addition of amines in aqueous solution and (**b**) surface treatment of the film with amines.

The results of the change in electrical resistance for the first option, where amines were added to the original PEDOT:PSS solution before the deposition of the films, are shown in Figure 11a. The addition of 1–7 ppm amines results in a 50% reduction in resistance due to the neutralization of the sulfamines in PEDOT:PSS. At 8 ppm, a sharp drop in resistance occurs, and then it is determined by the resistance of only PEDOT bound to polystyrene aminosulfate.

The effect of the introduction of amines was further enhanced by the addition of carbon nanotubes to the solution. Due to the porosity of carbon nanotubes and carbon radicals in amines, their reliable bonding is ensured. At the same time, the amino group remains free and can bind to the sulfo group. As a result, the conductivity of the entire system increases due to the conductivity of carbon nanotubes. The combined addition of amines with nanotubes at concentrations up to 7 ppm has little effect on the optical and mechanical properties of PEDOT:PSS. Each addition leads to the formation of fibrillar particles in the films, and their combination only enhances the fibrillar nature and aggregation as compounds. The results show that the additives directly affect the electrical properties of PEDOT:PSS when self-assembled during the hydrated film solidification phase that occurs within tens of seconds after centrifugation and annealing.

Now let us consider the second option when the surface of the freshly prepared film was dried at 105 °C. Surface modifiers based on polymers containing simple aliphatic amino groups can significantly reduce the work function of conductors and conducting polymers, including PEDOT:PSS. The decrease occurs due to the physical sorption of the neutral polymer, which converts the modified conductors into efficient electron-selective electrodes into organic optoelectronic devices. These polymer surface modifiers are treated in air from solution to obtain highly conductive materials as electrodes [36].

Conductivity behaves differently during surface treatment followed by heat treatment in air. The change in the resistivity of the PEDOT:PSS film, in this case, is shown in Figure 11b; with an increase in concentration, it falls by more than 20 times. Films with such low resistance are interesting for photovoltaic applications.

The experimental results showed that the doping/treatment of PEDOT:PSS films with aniline with 0.1% carbon nanotubes are the most effective way to reduce the electrical resistance of the PEDOT:PSS p-layer. Thus, the proposed liquid-phase methods for creating PEDOT:PSS composite layers using amines make it possible to improve their conductivity in a simple way and thereby increase the efficiency of photovoltaic cells.

## 4. Conclusions

By using methylammonium lead iodide as an example, a two-stage method for the formation of films of organometallic halide perovskites is proposed, which makes it possible to form films with large, densely packed crystallites. As a result, films of methylammonium lead iodide with crystallite sizes up to 500 µm were obtained, as well as films with the addition of low-walled carbon nanotubes. It was shown that nanotubes are embedded in the intercrystalline space, thereby facilitating the formation of films with no defects or punctures short-circuiting the electrodes.

Polymer films poly (3,4-ethylene dioxythiophene):polystyrene sulfonated acid (PEDOT:PSS) doped with a number of amines (aniline, aniline with 0.1%carbon nanotubes, Diethylamine, Octylamine, 1-Pentylamine, Methylamine) were obtained. Depending on the concentration of dopants, the electrical conductivity of PEDOT:PSS films can be significantly improved. Moreover, in the case of treatment with aniline, the transmission of the PEDOT:PSS films slightly increases due to bandgap narrowing. A variant of improving the conductivity by treating the surface of the finished film with the indicated amines followed by their heat treatment, which provides the best results, is considered. The greatest influence on the resistance drop is observed by doping/treatment with aniline. The experimental results showed that the treatment of PEDOT:PSS films with aniline with carbon nanotubes are the most effective way to increase the conductivity of the PEDOT:PSS p-layer.
